# Preliminary insights into patient preparedness for knee or hip arthroplasty: a descriptive survey study

**DOI:** 10.1186/s13104-023-06329-8

**Published:** 2023-04-24

**Authors:** Justine M Naylor, Ian A Harris, Sidhant Joon, Robert Boland, Bernadette Brady, Shaniya Ogul, Rajat Mittal

**Affiliations:** 1 Whitlam Orthopaedic Research Centre, Ingham Institute for Applied Medical Research, Liverpool, 2 Campbell St, Liverpool, NSW 2170 Australia; 2 School of Clinical Medicine, South West Sydney Clinical School, Faculty of Medicine and Health, UNSW Medicine & Health, Liverpool Hospital, NSW Sydney, Australia; 3 Orthopaedic Department, Liverpool Hospital, Locked Bag 7103, Liverpool, BC, NSW 1871 Australia; 4 Fairfield Hospital, Cnr Polding St and Prairievale Rd, Prairiewood, NSW 2176 Australia; 5 Faculty of Medicine and Health, University of Sydney, Sydney, NSW 2006 Australia; 6 Pain Clinic, Liverpool Hospital, Locked Bag 7103, Liverpool, NSW 1871 Australia

**Keywords:** Arthroplasty, knee, Arthroplasty, hip, Pre-operative education, Patient education

## Abstract

**Objective:**

Knowledge-based preparedness for surgery is achieved through education. It is unclear which of brief or extended education programs prior to knee or hip arthroplasty provides better patient preparedness. Using the Patient Preparedness for Surgery survey, we investigated whether people awaiting arthroplasty attending a hospital that provided education over multiple visits via a pre-surgery management program (‘Extended’) report superior preparedness compared to those attending a hospital in the same health district that only provides education at the pre-admission clinic assessment (‘Brief’).

**Results:**

A consecutive sample of 128 people (n = 101, ‘Extended’, n = 27 ‘Brief’) completed the anonymized survey. COVID-19 related service disruptions undermined the sample size, reducing statistical power. The pre-specified superiority of the Extended program (a relative 20% more reporting ‘agree’/’strongly agree’) was not observed for ‘Overall preparedness’ [95% (Extended) vs. 89% (Brief), p = 0.36]. Between-group differences exceeding 20% relative superiority were observed for three preparedness sub-domains [‘Alternatives explained’ (52 vs. 33%, p = 0.09); ‘Prepared for home’ (85 vs. 57%, p < 0.01); ‘Recall of complications’ (42 vs 26%, p = 0.14)]. The preliminary findings suggest an extended education program potentially yields better patient-reported preparedness in some preparedness sub-domains, but not all.

**Supplementary Information:**

The online version contains supplementary material available at 10.1186/s13104-023-06329-8.

## Introduction

Patient preparedness for elective surgery can help manage risks for the facility or care provider as well as promote better patient outcomes [1–4]. Fundamental to preparing patients is pre-operative education for the purposes of improving knowledge, and health behaviours and outcomes [5]. Prior to total knee or hip arthroplasty (TKA, THA), preoperative education classes have been shown to reduce patient anxiety, acute hospital length of stay, referral to inpatient rehabilitation and acute post-operative pain [6–10]. Some form of pre-operative education is considered key pre-arthroplasty [11–15], but there is no context-specific high-level evidence available to inform culturally sensitive, multi-language education approaches. Fundamental to an effective education program intended to comprehensively prepare people for major surgery is that it (i) is designed with end-users; (ii) is culturally sensitive, and (iii) can be understood by the majority both considering language and literacy levels. It is possible that programs that are informed by patients with lived experiences and cater for low-level literacy and varying cultural and language groups, may deliver greater and more consistent benefits. They may also reduce risk for the facilities and care providers if patients avoid complications or are less likely to make complaints about care received. The lack of co-design and cultural appropriateness of existing programs may explain (in part) why the benefits of pre-operative education are modest and that they have not been shown to reduce complications, patient complaints, or longer-term recovery [5, 14, 16].

As part of preparatory work to develop a culturally-sensitive, preoperative education program, we conducted a brief questionnaire survey of people undergoing TKA or THA at two arthroplasty facilities within South West Sydney - a region of vast cultural and ethnic diversity, and high-level socioeconomic disadvantage [https://abs.gov.au/census/find-census-data/quickstats/2021/127]. Due to differing service-level investment in arthroplasty services, the two facilities offer different pre-operative education programs, providing a ‘natural experiment’ opportunity to compare the programs in terms of patient-perceived preparedness.

The overall objective of our study was to determine if patient preparedness for surgery (education about surgery and the experience) is superior at a hospital which runs an extended compared to brief program. Specifically, we hypothesized that patients subjected to an extended program have more opportunity to interface with care providers and educators to clarify any uncertainties, and therefore will report higher levels of preparedness overall as well as for all aspects of preparedness subdomains.

## Materials and methods

### Design, setting and ethical approval

A cross-sectional, anonymized survey was used to capture participant responses at one time point pre-surgery. The two largest public hospitals within the South West Sydney Local Health District providing arthroplasty services were the sites involved. Both hospitals prior to COVID-19 were considered high (> 500 procedures per year, Hospital 1) or moderate volume (~ 200 procedures per year, Hospital 2) arthroplasty centres. Consistent with the demographics of the region, both hospitals serve a high proportion of people from culturally and linguistically diverse (CALD) backgrounds. Typically, approximately 25% of arthroplasty recipients at these hospitals do not speak English.

At one hospital (Hospital 1), pre-operative education is provided from the time the individual is waitlisted for surgery, typically one year prior to surgery. The program involves two or more assessments across the waiting period, finishing within 4–6 weeks prior to surgery with a group education program delivered in different languages, and a pre-admission clinic assessment. Hospital 2 provides one-to-one education by trainee orthopaedic registrars and anaesthetists during a single, mandatory pre-admission clinic visit 4–6 weeks prior to surgery. Patients are invited to return the following day for further education by nursing and allied health (physiotherapy and/or occupational therapy) staff, but attendance is not compulsory. Both programs cover education relating to immediate preparation for surgery, the acute care period, and subacute period with interpreters used as required. Hospital 1 also covers alternatives to surgery as well as advice regarding weight management and physical activity. Neither program was co-designed with end-users representing all cultural backgrounds and thus are not considered to be culturally sensitive.

The study was approved by the South West Sydney Local Health District Human Research Ethics Committee, following the Low or Negligible Risk Review Pathway (2021/ETH11241).

### Participant screening and eligibility

Screening for eligibility was undertaken by pre-admission nursing staff. People ≥ 18 years old waitlisted for primary total knee or hip arthroplasty secondary to osteoarthritis, who could understand English, Arabic, Greek, Simplified Chinese, or Vietnamese and who attended the pre-admission clinic at either hospital were eligible to complete the survey. People with documented dementia were excluded as were people who did not attend the nursing/allied health clinic at Hospital 2. Once screening was established, a research officer gave eligible people an information sheet in a relevant language detailing the purpose of the survey. Those who were willing to participate completed the survey anonymously in the relevant language, returning it to the research officer so service providers were not privy to the responses. As approved by the health district’s ethics committee, written consent was waived as completion of the anonymous survey implied consent. People who were eligible, but unable to complete the survey on the day were given the information sheet and contacted later by telephone within a week of the clinic visit.

### Data collection procedure

The Patient Preparedness for Surgery questionnaire, adapted from Kenton et al. [1] and Greene et al. [3], was used. It comprised nine Likert-type statements, each having five responses requiring respondents to rate how strongly they agreed/disagreed with the statement (Additional file 1). The survey covered the following education elements relevant to becoming ready for surgery - alternatives to, and purpose and benefits of, surgery; risks, benefits and complications of surgery; preparedness about acute and post-discharge experiences, and; overall preparedness. In addition to asking respondents whether they ‘understood’ the complications from surgery, we added an additional question asking the respondents to list possible surgical complications they recalled being told about. This question served as a measure of actual recall about a specific element of the education.

To confirm or deny similarities in population characteristics between the cohorts, participants were also asked to provide basic demographic and medical data (Additional file 1).

### Outcomes

The primary outcome was ‘Overall preparedness’. Secondary outcomes included the remaining eight Likert statements as well as the type and number of complications recalled. For analysis, the proportion in each group reporting ‘Agree’/’Strongly agree’ was calculated for all Likert statements.

### Sample size and data analysis

In the absence of prior studies capturing patient preparedness for arthroplasty surgery using a specific preparedness survey, we deferred to studies involving patients undergoing reconstructive pelvic surgery using the same survey to inform our sample size calculation [1, 17]. In these studies, > 95% agreed/strongly agreed they were prepared overall prior to surgery. Using a more conservative estimate of overall preparedness, a sample of 261 respondents (n = 174 Hospital 1, n = 87 Hospital 2) was required to provide 80% power (alpha 0.05) to detect a ~ 20% relative superiority in the proportion reporting Agree/Strongly agree for overall preparedness at Hospital 1 over Hospital 2 (85% vs. 70%). We hypothesized that the hospital with the extended program would have better results for all surveyed elements. Over a 6-month recruiting period, based on pre-COVID-19 rates of surgery, a sample of approximately 350 people was anticipated. The larger sample would permit secondary multivariable regression analysis of the primary outcome.

Descriptive statistics [mean, standard deviation, frequencies (%)] and independent t-tests or χ^2^ tests were used to report and compare the characteristics of the two cohorts. For analysis of outcomes, all between-group differences in proportions were analysed using the χ^2^ test or Fisher’s exact test of independence. Secondary multivariable regression analysis was planned to determine if other variables along with hospital (program type) were significantly associated with the primary outcome. Data were collated in Microsoft Excel and analysed using IBM SPSS Version 26.

## Results

### Impact of COVID-19 and cohort ascertainment

Continued COVID-19 related disruptions to public hospital surgical services and staffing, and a State-wide waitlist reduction strategy for publicly insured patients to be operated upon in the private sector, led to a slower than expected study recruitment rate. The study was prematurely terminated in view of anticipated continued disruptions.

**Fig. 1:**
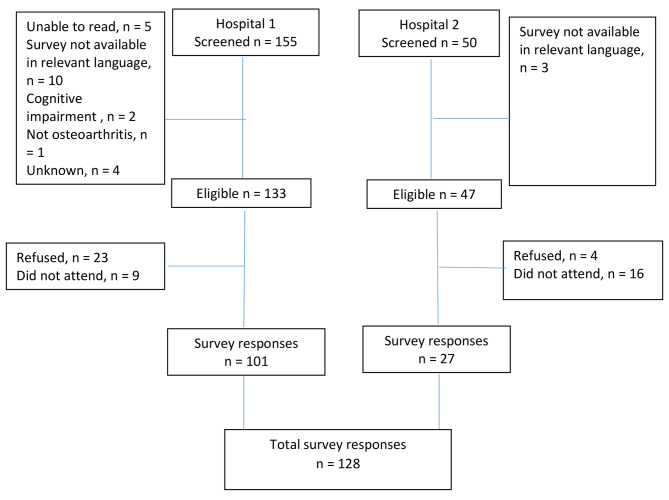
Cohort ascertainment

Over a 6-month period (February –August 2022), 205 people were screened, 25 were ineligible and 128 people completed the survey (Fig. 1).

### Cohort characteristics

The two cohorts manifested similar demographic characteristics except that Hospital 1 had more females (Table 1). The proportion who spoke English was high in both groups (89%).

**Table 1:** Characteristics of the hospital-based samples

	Hospital 1 Extended program n = 101	Hospital 2 Brief program n = 27	P-Value
Age, yr (mean, sd)	68.7 (8.8)	69.7 (9.5)	0.61
Female, %	51	30	0.05
Awaiting TKA, %	61	69	0.44
English-speaking, %	89	89	1.0
Australian-born, %	49	41	0.45
Education, %			0.09
No school	1	11	
Primary only	11	15	
Junior high school	51	37	
Completed school	24	22	
Tertiary	14	15	
Previous TKA/THA, %	25	22	0.79
Heart disease, %	15	26	0.25
Lung disease, %	4	7	0.61
Prior CVA/TIA, %	2	0	1.0
Renal failure, %	3	4	1.0
Past/current cancer, %	18	15	1.0

Key: Percentages rounded

### Outcomes

A high proportion in each hospital reported they Agreed/Strongly agreed that they were prepared overall (95% Hospital 1 vs. 89% Hospital 2, p = 0.36) (Table 2). The proportions in Hospital 1 reporting they Agreed/Strongly agreed exceeded the superiority margin for ‘Knowing the alternatives to surgery’ (52 vs. 33%, p = 0.09), the ‘Recall of any complications’ (42 vs. 26%, p = 0.14), and ‘Prepared for what to expect at home’ (85 vs 57%, p < 0.01), but the difference only reached statistical significance for the latter. For all other preparedness subdomains, between-hospital proportions reporting Agree/Strongly agree were similar, though for most subdomains, the proportion favoured the Extended program. Likewise, the recall of specific complications was generally similar though more (greater variety) of complications were recalled by the Hospital 1 cohort.

**Table 2:** Preparedness survey outcomes

	Hospital 1 Extended program N = 101	Hospital 2 Brief program N = 27	P-Value
Overall, I feel prepared, %	95	89	0.36
Pre-operative preparedness			
Know about alternatives to surgery, %	52	33	0.09
Understand purpose of surgery, %	94	96	1.0
Understand benefits of surgery, %	94	93	0.68
Understand risks of surgery, %	82	74	0.35
Understand complications of surgery, %	80	78	0.78
Complications recalled, %	42	26	0.14
Infection	16	15	1.0
Pain	6	7	0.68
Cardiovascular issues	6	4	1.0
Nerve damage	8	0	0.20
Stroke	2	0	1.0
Death	3	4	1.0
Do not want to know	1	4	0.38
Stiffness or swelling	2	0	1.0
Blood clot	6	0	0.34
Post-operative preparedness			
I feel prepared for what to expect in hospital, %	88	82	0.35
I feel prepared for what to expect when I am home, %	85	56	**<** 0.01
Doctors and nurses have spent enough time preparing me for my upcoming surgery, %	94	82	0.05

Key: Percentages rounded

## Discussion

Notwithstanding the smaller than expected sample, our study provides unique and actionable insights for research concerning patient preparedness for surgery.

Despite neither hospital-based program being co-designed nor culturally informed, and despite overt differences in the delivery of patient education, overall preparedness, and indeed several of the subdomains, at least as perceived by patients, was and were very high. Interestingly, the high overall rates are consistent with previous research involving pelvic reconstructive surgery [1, 17]. On a simplistic level, it is tempting to conclude that comprehensive, culturally appropriate programs are not required to obtain high-level patient preparedness. However, we caution against this simplistic interpretation for a few reasons and contend that better or differing methodologies may be required in order to ascertain preparedness for surgery.

Firstly, reference to the sub-domains where the two cohorts did appear to differ (ignoring the lack of statistical power to confirm most differences), reveal a more nuanced picture whereby it seems the more extensive (more detailed) program may yield higher levels of perceived preparedness for surgery in some areas which is in keeping with our hypotheses. What we cannot appreciate from our study is whether better preparedness in these areas translated to meaningful differences in clinical outcomes and recovery. So, at the very least, concurrent measurement of clinical or service outcomes alongside preparedness measures are likely required if we want to quantify or understand the transactional value of preparedness. Secondly, that the recall of specific complications was overwhelmingly poor despite responders in both groups generally agreeing they understood the risks and complications of surgery, presents a significant conundrum for service providers and/or researchers. Is the discrepancy due to responders merely providing answers they think the researcher or care provider wants (‘Yes we understand what you have told us’), but when challenged, we can see the responder does not really understand. Or, does the responder truly think they understand (for example, ‘I understand that there is a risk of complications’), but the specifics of what is being imparted (in this case, complication type) is not important to them or worse still, not wanted [18]. Going forward, incorporating measures of understanding of what is taught in order to improve preparedness may reveal associations between understanding and post-operative outcomes that are otherwise overlooked when preparedness alone is the construct under scrutiny.

### Limitations

The small sample size prevented definitive conclusions concerning the superiority of the extended program and examination of potential confounders of preparedness such as the ability to speak English, education level, and previous exposure to arthroplasty. The low proportion of non-English speakers within both cohorts (likely indicating a recruitment bias) means the results may not reflect the perceptions of this sub-population. Assessment of preparedness pre-operatively may not reflect patient perception of preparedness at a later time. That said, if anxiety reduction pre-surgery is a target for preparedness programs, pre-operative measurement arguably remains more pertinent.

## Electronic supplementary material

Below is the link to the electronic supplementary material.

Supplementary Material 1: Additional file 1: Preparedness Survey, Additional file 2: Survey data

## Data Availability

All data generated or analysed during this study are available from the corresponding author on reasonable request. ***Competing interests***. Nil to declare.

## Abbreviations

TKA: total knee arthroplasty
THA: total hip arthroplasty
