# Supplementary material for: Preliminary insights into patient preparedness for knee or hip arthroplasty: a descriptive survey study
Source: BMC Res Notes. 2023 Apr 24;16:60. doi: 10.1186/s13104-023-06329-8 (PMC10123575; doi:10.1186/s13104-023-06329-8)
Supplement: Supplementary file 1 — Supplementary Material 1: Additional file 1: Preparedness Survey, Additional file 2: Survey data [file 13104_2023_6329_MOESM1_ESM.docx]

| **PATIENT PREPAREDNESS FOR SURGERY** | | | | | | | | | | | |
| --- | --- | --- | --- | --- | --- | --- | --- | --- | --- | --- | --- |
| Age (years)______________ | Sex (tick): Male Female | | Hospital (tick): Fairfield Bankstown | | | | | Country of birth:__________________ | | | |
| Knee replacement Hip replacement | | Do you speak English? Yes No | | | | Primary language spoken at home: | | | | | |
| Education level (tick):  No school Primary Only Completed junior high school (Yrs 9/10) Completed Senior High School (Yr 11/12) Tertiary/University Degree | | | | | | | | | | | |
| Medical Health (tick if Yes): Heart disease Lung disease Previous stroke/CVA/TIA Renal/kidney failure  Past/Current cancer Previous knee or hip replacement | | | | | | | | | | | |
| For each statement tick the box that matches your level of agreement regarding your upcoming surgery | | | | | | | | | | | |
|  | | | | Strongly Agree | Agree | | Somewhat Agree | | Somewhat Disagree | Disagree | Strongly Disagree |
| I know about the *alternatives* to the planned surgery | | | |  |  | |  | |  |  |  |
| I understand the *purpose* of the planned surgery (what this surgery can accomplish). | | | |  |  | |  | |  |  |  |
| I understand the *benefits* of the planned surgery (how this surgery should help me). | | | |  |  | |  | |  |  |  |
| I understand the *risks* of the planned surgery (what the chances are of something not going the way my doctor and I want it to go). | | | |  |  | |  | |  |  |  |
| I understand the *complications* of the planned surgery (what problems can come from this surgery). | | | |  |  | |  | |  |  |  |
| Please write in the space below what complications you have been told about / you are aware of | | | | | | | | | | | |
|  | | | | | | | | | | | |
| I feel prepared about what to expect after surgery *while I am in the hospital*. | | | |  |  | |  | |  |  |  |
| I feel prepared about what to expect after surgery *when I am at home*. | | | |  |  | |  | |  |  |  |
| My doctors and nurses have spent enough time preparing me for my upcoming surgery. | | | |  |  | |  | |  |  |  |
| Overall, I feel prepared for my upcoming surgery | | | |  |  | |  | |  |  |  |
| **Adapted from *Kenton et al 2007* and *Kristie et al 2017*** | | | | | | | | | | | |

ADDITIONAL FILE 1 – Naylor et al, Preliminary insights into patient preparedness for knee or hip arthroplasty: a descriptive survey study
